# Effects of stabilizer magnesium nirate on CMIT/MIT-induced respiratory toxicity

**DOI:** 10.1007/s43188-023-00170-8

**Published:** 2023-03-10

**Authors:** Mi-Kyung Song, Yong-Wook Baek, Dong Im Kim, Sung-Hoon Yoon, Kyuhong Lee

**Affiliations:** 1 Inhalation Toxicology Center for Airborne Risk Factor, Korea Institute of Toxicology, 30 Baekhak1-Gil, Jeongeup, Jeollabuk-Do, 56212 Republic of Korea; 2 Department of Human and Environmental Toxicology, University of Science and Technology, Daejeon, 34113 Republic of Korea; 3 Humidifier Disinfectant Health Center, Jeongeup, Republic of Korea; 4 Humidifier Disinfectant Health Center, Environmental Health Research Department, National Institute of Environmental Research, Hwangyong-Ro 42, Seogu, Incheon, 22689 Republic of Korea

**Keywords:** Humidifier disinfectant, Chloromethylisothiazolinone, Methylisothiazolinone, Stabilizer, Magnesium nitrate, Lung injury

## Abstract

Despite a humidifier disinfectant (HD) product containing chloromethylisothiazolinone (CMIT) and methylisothiazolinone (MIT) with approximately 22% magnesium nitrate as a stabilizer, no report on the effects of magnesium nitrate on the respiratory toxicity of CMIT/MIT is available. In this study, Kathon CG and Proclin 200, containing approximately 1.5% CMIT/MIT with different magnesium nitrate concentrations (22.6% and 3%, respectively), were used to compare respiratory effects after intratracheal instillation (ITI) in C57BL/6 mice. C57BL/6 mice were randomized into groups of saline control, magnesium nitrate, Kathon CG, and Proclin 200 with 1.14 mg/kg of CMIT/MIT as the active ingredient, and administration was performed 6 times in a 2–3 day-interval in 2 weeks in all groups. Differential cell count analysis, cytokine analysis, and histological analysis of lung tissue were performed to characterize the injury features. Both Kathon and Proclin 200 induced an increase in inflammatory cell levels in the bronchoalveolar lavage (BAL) fluid, in particular, eosinophils and type 2 T helper cell (Th2)-secreted cytokines. All histopathological changes including granulomatous inflammation, mixed inflammatory cell infiltration, mucous cell hyperplasia, eosinophil infiltration, and pulmonary fibrosis were induced with similar frequency and severity in Kathon CG and Proclin 200 groups. Our results suggested that magnesium nitrate did not affect CMIT/MIT-induced lung injury in the intratracheally instilled model. Further inhalation studies are needed to determine the distribution and toxicity differences of CMIT/MIT in the lungs according to the magnesium nitrate concentration.

## Introduction

Humidifier disinfectants (HDs), which are dispersed into the air by the humidifier, were identified as a potential cause of an outbreak of lung injury in South Korea [1]. Chloromethylisothiazolinone (CMIT) and methylisothiazolinone (MIT), which are major components of HDs, are associated with fatal health effects such as lung injury [2]. Patients who were exposed to only a CMIT/MIT-containing HD product showed progressive respiratory difficulty, similar to humidifier disinfectant-associated lung injury (HDLI) caused by exposure to polyhexamethylene guanidine phosphate (PHMG-P) or oligo(2-(2-ethoxy)ethoxyethyl guanidinium chloride (PGH) [3]. Commercial products of CMIT/MIT are a 3:1 ratio mixture and have been widely used in water-based industrial products and cosmetics as preservatives. Until now, various types of isothiazolinone-containing products have been registered and used as chemical additives in shampoos, hair rinses, disposable hand disinfectants, cosmetics, and many more products [3, 4] (see also United States Environmental Protection Agency, USEPA 1998). Despite the safety concerns becoming much essential with the frequent usage of various types of isothiazolinone-containing products, the potentially harmful effects on human health, especially respiratory effects, were not yet identified in sufficient detail. Only a previous study in our research group revealed Kathon-induced fibrotic lung injury in a mouse model [5].

CMIT/MIT does not exist alone in HD products but is mixed with a significant amount of magnesium nitrate (22.6%), which is added as a stabilizer [3]. Magnesium nitrate is commonly used as a laboratory chemical, intermediate, and fertilizer. It is widely used as a stabilizer owing to its high preservative effect [6]. Although available data report that inhalation of magnesium nitrate may cause skin, eye, and respiratory irritation [7], experimental evidence for the effects of magnesium nitrate on the respiratory tract is insufficient. In addition, although the percentage of magnesium nitrate concentration (22.6%) is significantly higher than that of CMIT/MIT (approximately 1.5%), there are no reports on the effects of magnesium nitrate on respiratory distribution and toxicity of CMIT/MIT.

Several studies have reported the effects of stabilizing agents on the physicochemical properties and toxicity of active ingredients [8–10]. These studies have revealed the impact of stabilizers including anionic, cationic, and amphoteric surfactants and cationic polymers on the physicochemical properties, stability, and behavior of active ingredients as well as on toxicity.

To understand the toxicity of CMIT/MIT-containing HD products clearly, the effects of magnesium nitrate on the respiratory toxicity of CMIT/MIT need to be investigated.

This study aimed to evaluate the impact of differences in the concentrations of magnesium nitrate in two HD products containing CMIT/MIT on CMIT/MIT-induced lung injury. Kathon CG and Proclin 200 with similar CMIT/MIT concentrations but different magnesium nitrate concentrations (22.6% and 3%, respectively) were intratracheally instilled in C57BL/6 mice to compare the severity and pattern of respiratory injury caused by HD with a low magnesium nitrate concentration with that caused by HD with a high magnesium nitrate concentration.

A better understanding of the role of magnesium nitrate in the toxicity of CMIT/MIT-containing mixtures allows potential safety proposals related to the manufacture and use of CMIT/MIT-containing products.

## Materials and methods

### Animals and the study protocol

The experimental procedures were approved by the Institutional Animal Care and Use Committee of the Korea Institute of Toxicology (Number 21–01-0170). Thirty-five C57BL/6NCrlOri male mice (6-week-old) were obtained from Orient Bio Inc. (Seongnam, Korea). The mice were housed in polycarbonate cages (135W × 280L × 145H mm) in an environmentally controlled animal facility with 22 ± 3 °C controlled temperature, 50 ± 10% relative humidity, 150–300 Lux light intensity, and a 12 h light/dark cycle. The air ventilation system in the animal room was operational 10–20 times/h.

Based on the most recently measured body weight (BW), mice were randomly assigned to the vehicle control group, magnesium nitrate, Kathon, and Proclin 200 exposed group using the Pristima System (Version 6.4; Xybion Medical System Co., Lawrenceville, NJ, USA), a toxicology data management program. The study consisted of five groups defined by their specific exposure profile (each group, *n* = 7): a vehicle control group, two magnesium nitrate exposed groups of 3% and 22.6% concentrations, a Kathon and a Proclin 200 exposed groups of 1.14 mg active ingredient (a.i.)/kg dose.

### Kathon and Proclin 200 instillation

The constituents of CMIT/MIT-containing products are shown in Table 1. The concentrated stock solution of Kathon CG (1.519% CMIT/MIT, 22.6% magnesium nitrate from DOW Chemical Com., Midland, MI, USA) and Proclin 200 (1.5% CMIT/MIT, 3% magnesium nitrate from Sigma-Aldrich, St. Louis, MO, USA) was diluted in saline to create equivalent doses of 1.14 mg a.i./kg, which were intratracheally instilled using a modified automatic video instillator (Doobae System, Seoul, Korea) at 50 μL/head. The dose of magnesium nitrate was calculated based on the amount of magnesium nitrate contained therein after the dilution of Kathon CG and Proclin 200. All substances including saline, magnesium nitrate, Kathon CG, and Proclin 200 were administered six times on days 1, 4, 6, 8, 11, and 13. Before the intratracheal instillation (ITI), the mice were exposed to 2.5% isoflurane and instillation was performed immediately.

**Table 1:** Constituents of CMIT/MIT-containing products

Composition	Kathon CG	Proclin 200
Active ingredient (CMIT/MIT)	1.519%	1.5%
Matrix	Water	Water
Stabilizer	22.6% Mg nitrate	3% Mg nitrate

Twenty-four hours after the last ITI dosing, the mice were euthanized by continuous exposure to an overdose of isoflurane until one minute after they stopped breathing. Weight of the left lung was recorded, and the lung samples were fixed in 10% formalin or stored in a -70 °C deep freezer until analysis.

### Bronchoalveolar lavage fluid (BALF) analysis

After the mice were anesthetized, the left lungs were ligated, and the right lungs were gently lavaged thrice via a tracheal tube using phosphate-buffered saline (PBS, Thermo Fisher Scientific Inc., Waltham, MA, USA). Using a NucleoCounter (NC-250; ChemoMetec, Gydevang, Denmark), the total cells of the collected BALF were counted. For differential cell counts, BALF cell smears were prepared using Cytospin (Thermo Fisher Scientific Inc.) and stained with Diff-Quik solution (Dade Diagnostics, Aguada, Puerto Rico). The different cell types were counted (*n* = 200/slide). BALF was immediately centrifuged at 452 rcf for 5 min, and the collected supernatant was stored at – 70 ℃ until the cytokine levels were measured through enzyme-linked immunosorbent assay (ELISA). Interleukin (IL)-4, IL-5, IL-6, IL-13, and tumor necrosis factor-alpha (TNF-α) were quantified through ELISA using commercial kits (Thermo Fisher Scientific Inc.), according to the manufacturer’s protocol.

### Histopathology analysis

The left lung tissues were fixed in 10% neutral-buffered formalin and staining was performed at Korean Pathology Technical Center (KP&T, Cheongju, Korea). Briefly, the paraffin-embedded tissue blocks were cut into 4 μm sections and stained with Hematoxylin & Eosin (H&E), Periodic acid-Schiff (PAS), and Masson’s trichrome (MT) (Sigma-Aldrich, St. Louis, MO, USA) solutions. Stained sections were analyzed under a light microscope (Axio Imager M1; Carl Zeiss, Oberkochen, Germany). Each successive field was individually assessed to determine the severity of inflammatory cell infiltration, eosinophils infiltration to perivascular/alveolar areas, mucus production, and goblet cell hyperplasia. The lesions were estimated by an experienced histopathologist using a blinded, scoring system outlined previously [11, 12]. The degree of histological observations was graded on a semi-quantitative scale of 0 to 4 depending on the degree–0 grade indicated that the tissue was normal or showed no changes and grades were assigned as 1 (minimal), 2 (slight), 3 (moderate), or 4 (severe) based on an increasing severity or complexity of changes.

### Statistical analysis

All data were presented as the mean ± standard deviation (SD). Statistical analysis was performed using GraphPad Prism software (v8, GraphPad Software Inc., San Diego, CA, USA). Statistical multiple comparisons were performed by one-way analysis of variance followed by Dunnett’s test. Differences were considered significant at ^*^p < 0.05, ^**^p < 0.01, ^***^p < 0.001, and ^****^p < 0.0001.

## Results

### BW and BW gain changes

**Fig. 1:**
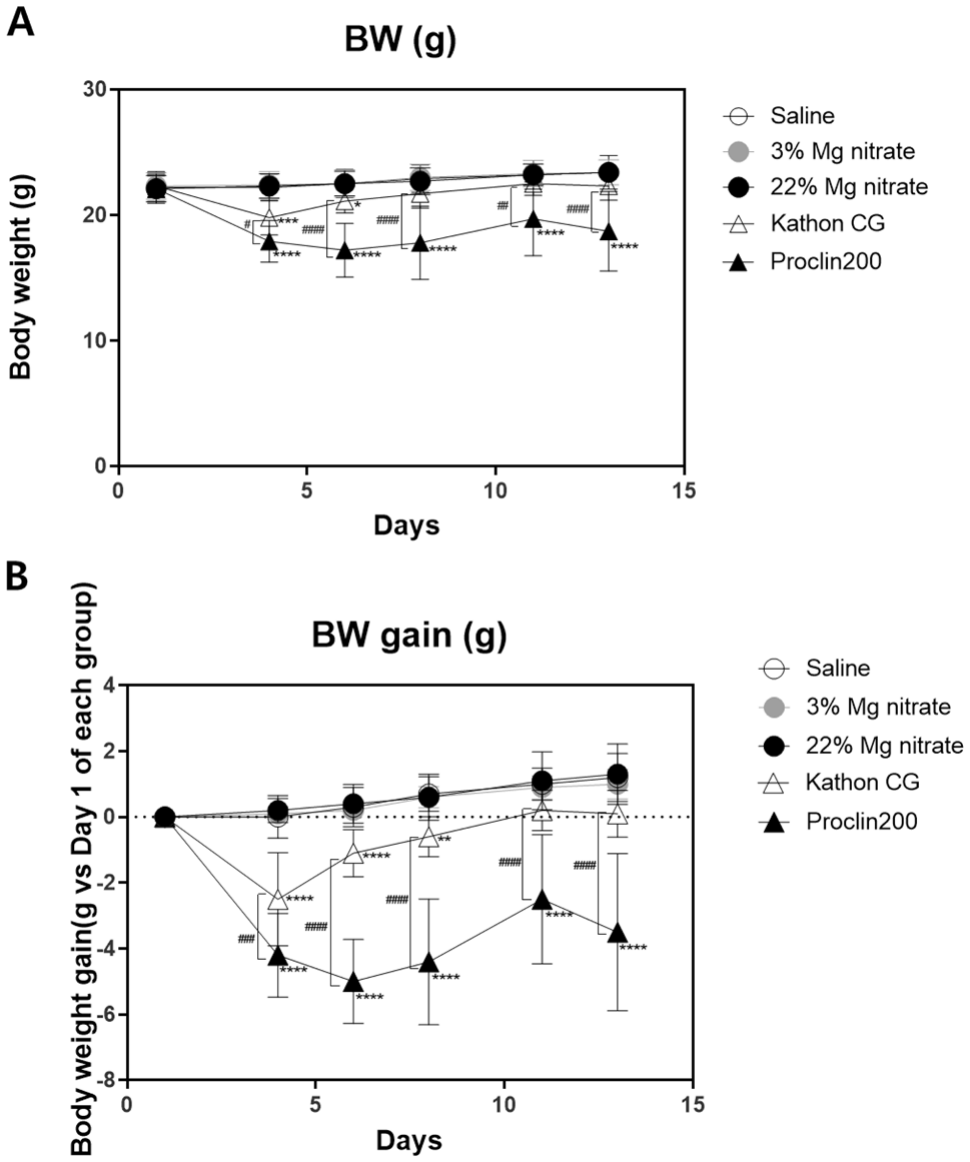
Changes in the BW and BW gain of mice. Changes in the BW of mice during the experimental period (**a**) and changes in the BW gain of mice during the experimental period relative to Day 1 (**b**). The results were expressed as the mean ± SD for each group. ^*^p < 0.05, ^**^p < 0.01, ^***^p < 0.001 and ^****^p < 0.0001 vs. the saline control group, ^*#*^*p* < 0.05, ^*##*^*p* < 0.01, ^*###*^*p* < 0.001 and ^*####*^*p* < 0.0001 vs. the Kathon CG group

BWs were measured a total of six times throughout the administration period (Days 1, 4, 6, 8, 11, and 13), and changes in weight relative to Day 1 were monitored. There were no significant changes in the BW of mice in the saline and magnesium nitrate-instilled groups during the experimental period. However, all the mice that were intratracheally instilled with Kathon CG and Proclin 200 showed significant weight change relative to the control group throughout the administration period (Fig. 1A). Similar to the changes in BW, the changes in the BW gains of mice in the Kathon CG and Proclin 200-instilled groups were significantly decreased (Fig. 1B). Changes in BW and BW gain were greater in the Procline 200-instilled group than in the Kathon CG-instilled group, and a significant difference was observed between the two groups.

### General symptoms and gross examination

There were no remarkable general symptoms and gross pathology in the saline and magnesium nitrate-instilled groups, but irregular respiration, wheezing, and emaciation were observed in the Kathon CG and Proclin 200-instilled groups. Additionally, all the mice that received Kathon CG and Proclin 200 showed red or pale discoloration of the lungs.

### Organ weight changes

**Fig. 2:**
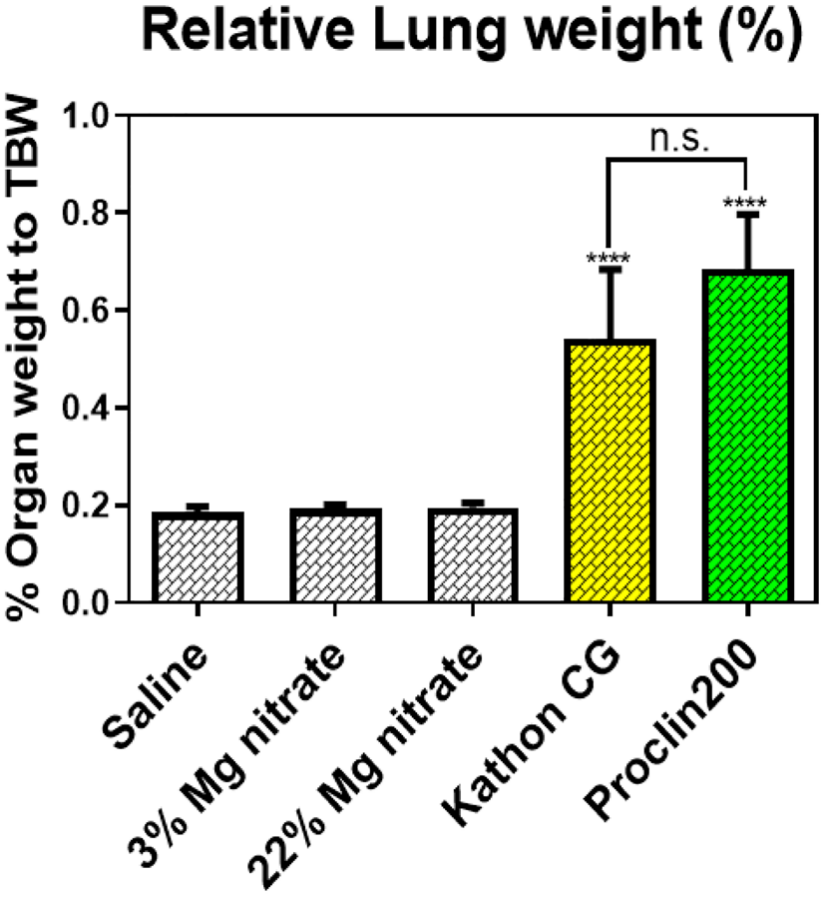
Changes in the relative lung weights of the saline, magnesium nitrate, Kathon CG and Proclin 200-instilled mice groups. The relative lung weights were calculated using the following formula: relative organ weight = organ weight (g)/terminal body weight (g) × 100%. The results were expressed as the mean ± SD for each group. ^****^p < 0.0001 vs. the saline control group. n.s. = not statistically significant

The lung weight of mice in the Kathon CG or Proclin 200-instilled groups increased significantly compared to the saline control group (Fig. 2). The level of change in the weight of the lung was similar between the mice that received Kathon CG and Proclin 200. There were no differences in lung weight in mice in the saline and magnesium nitrate-instilled groups.

### Histopathological examination

**Fig. 3:**
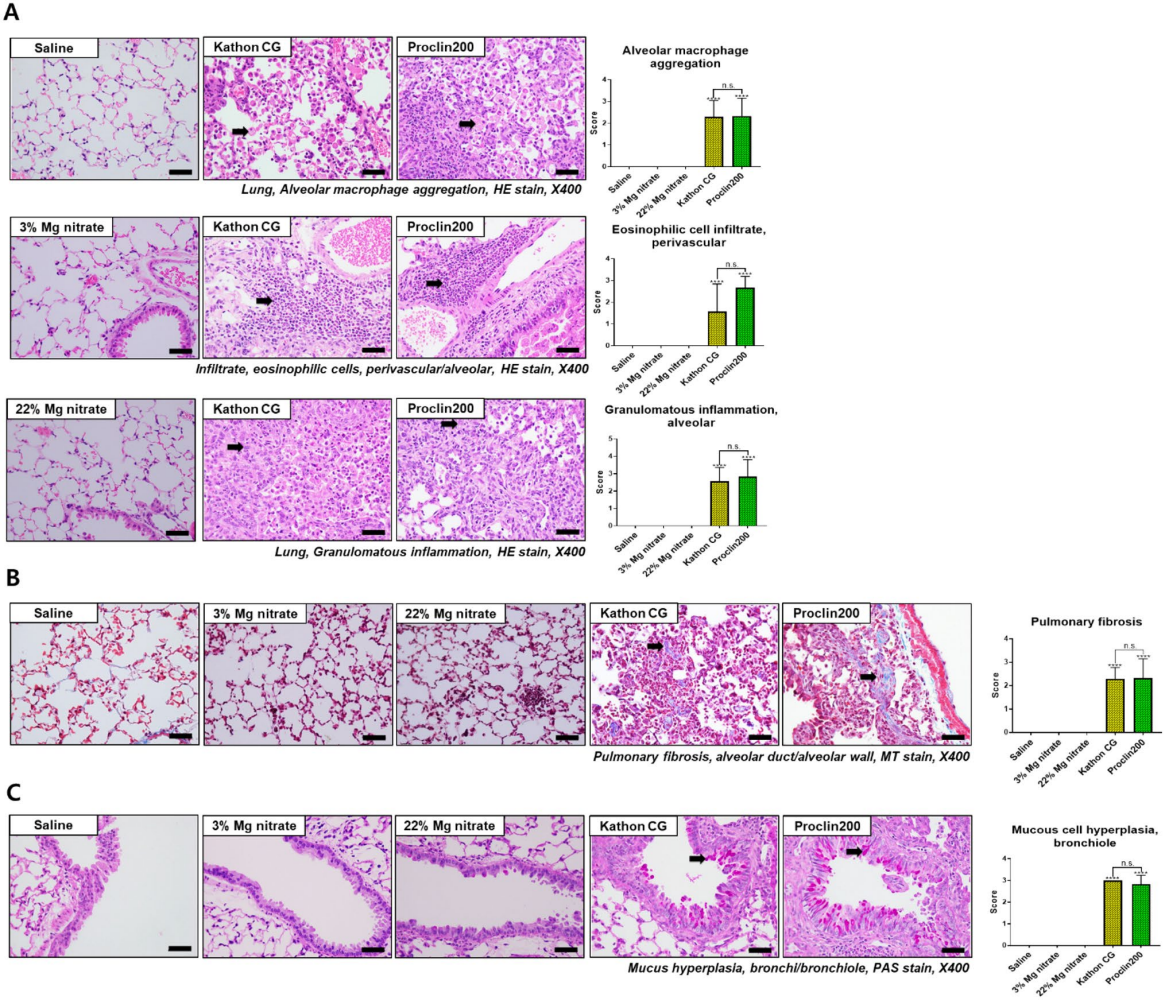
Representative H&E-stained lung sections and the inflammation scores of alveolar macrophage aggregation, eosinophils infiltration, and granulomatous inflammation (**a**). Representative MT-stained sections of the lung and the inflammation scores of pulmonary fibrosis (**b**). Representative PAS-stained lung sect. ions and the inflammation scores of mucous cell hyperplasia (**c**). Black arrows indicate inflammatory cells, collagen, and goblet cell deposition. Scale bar = 200 µm. The results were expressed as the mean ± SD for each group. ^****^p < 0.0001 vs. the saline control group. n.s., not statistically significant

Histological analysis was performed to identify the pathological features of Kathon CG- or Proclin 200-induced lung injury by H&E, PAS, and MT staining. There were no significant histological changes in the lungs of the saline and magnesium nitrate-instilled mice. On the other hand, various injury-associated histological findings were observed in the lungs of the Kathon CG- and Proclin 200-instilled mice. In H&E staining, alveolar macrophage aggregation, eosinophil infiltration in the perivascular site, and alveolar granulomatous inflammation increased markedly in the Kathon CG- and Proclin 200-instilled mice compared to control mice (Fig. 3A). MT staining revealed that collagen deposition was markedly elevated in the Kathon CG- and Proclin 200-instilled mice compared to control mice (Fig. 3B). According to the PAS staining results, mucous cell hyperplasia increased significantly in the Kathon CG- and Proclin 200-instilled mice compared to control mice (Fig. 3C). All histopathological changes including granulomatous inflammation, mixed inflammatory cell infiltration, mucous cell hyperplasia, eosinophil infiltration, and pulmonary fibrosis were induced with similar incidence and severity in both Kathon CG and Proclin 200 groups.

### BALF cell analysis

**Fig. 4:**
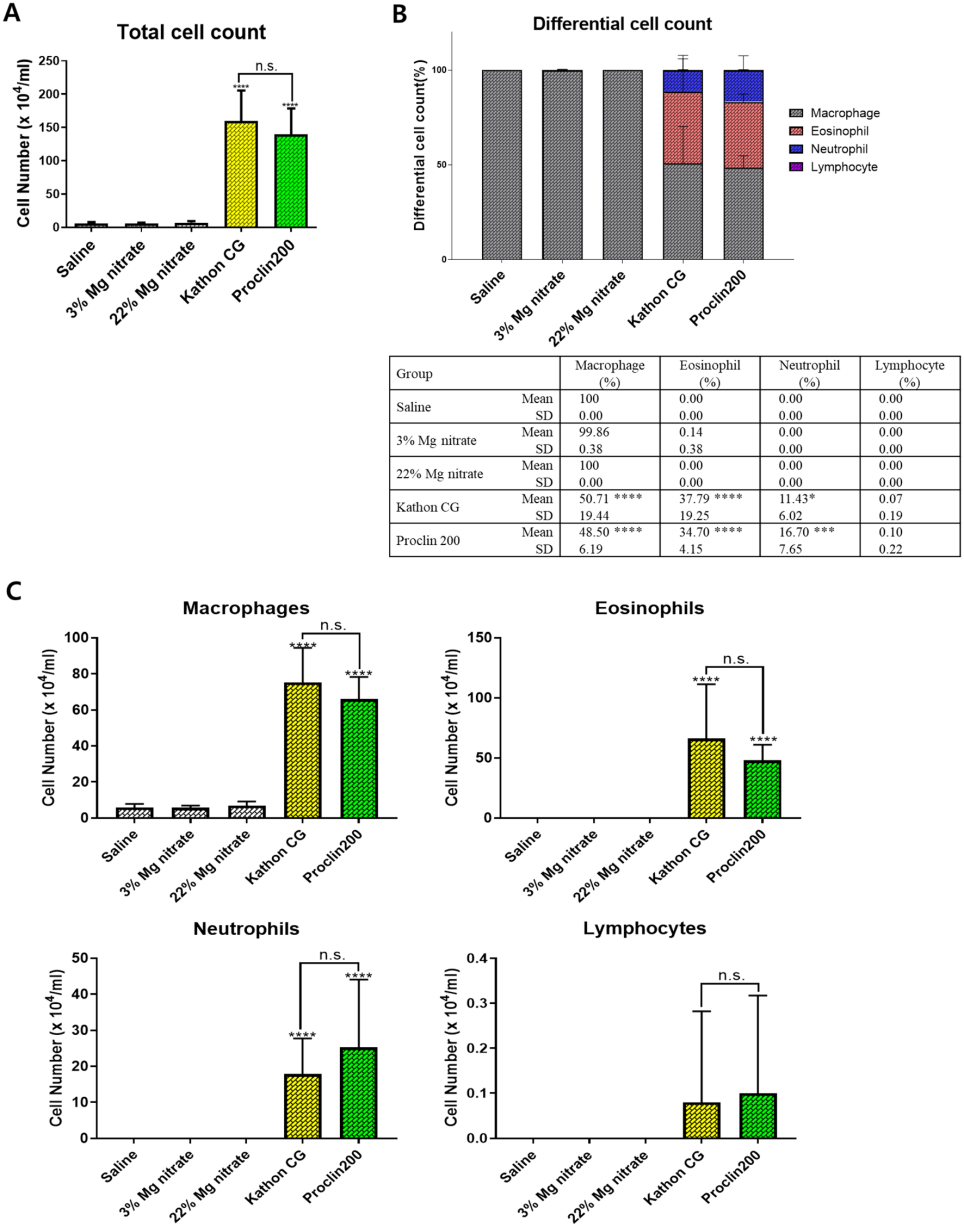
The effects of Kathon CG and Proclin 200-induced changes on total and differential cell counts in the BALF of mice. The total number of cells (**a**), cell population composition as a percentage of total cells (**b**), and the number of macrophages, eosinophils, neutrophils, and lymphocytes (**c**) in the BALF of saline, magnesium nitrate, Kathon CG, and Proclin 200-instilled mice. The results were expressed as the mean ± SD for each group. *p < 0.05, ***p < 0.001 and ****p < 0.0001 vs. the saline control group. n.s., not statistically significant

There were no significant cytological changes in the BALF of the saline- and magnesium nitrate-instilled mice. On the other hand, both Kathon CG and Proclin 200 groups showed a considerable increase in the total cell count. The proportion and number of inflammatory cells such as macrophages, eosinophils, and neutrophils were significantly changed (Fig. 4).

Moreover, a significant increase in the total number of cells was observed in the Kathon CG and Proclin 200 groups (Fig. 4A). Also, the proportion of eosinophils and neutrophils was considerably increased in the Kathon CG- and Proclin 200-instilled groups (Fig. 4B). In the Kathon CG and Proclin 200 groups, the increase in the proportion of eosinophils was dominant, with eosinophils comprising approximately 38% and 35% of the total BALF cells, respectively. The proportion of neutrophils significantly increased to approximately 11% and 17% of the total BALF cells in the Kathon CG and Proclin 200 groups, respectively. Additionally, a remarkable increase in the absolute number of macrophages, eosinophils, and neutrophils was observed in the Kathon CG and Proclin 200 groups (Fig. 4C). The cytological changes of inflammatory cells in both Kathon CG and Proclin 200 groups were similar.

### Cytokine levels in BALF

To investigate the release of inflammatory mediators induced by Kathon CG and Proclin 200 instillation, we evaluated the levels of several inflammatory cytokines such as T helper 2 (Th2)-secreted IL-4, IL-5, IL-13, and IL-6, and TNF-α in the BALF.

**Fig. 5:**
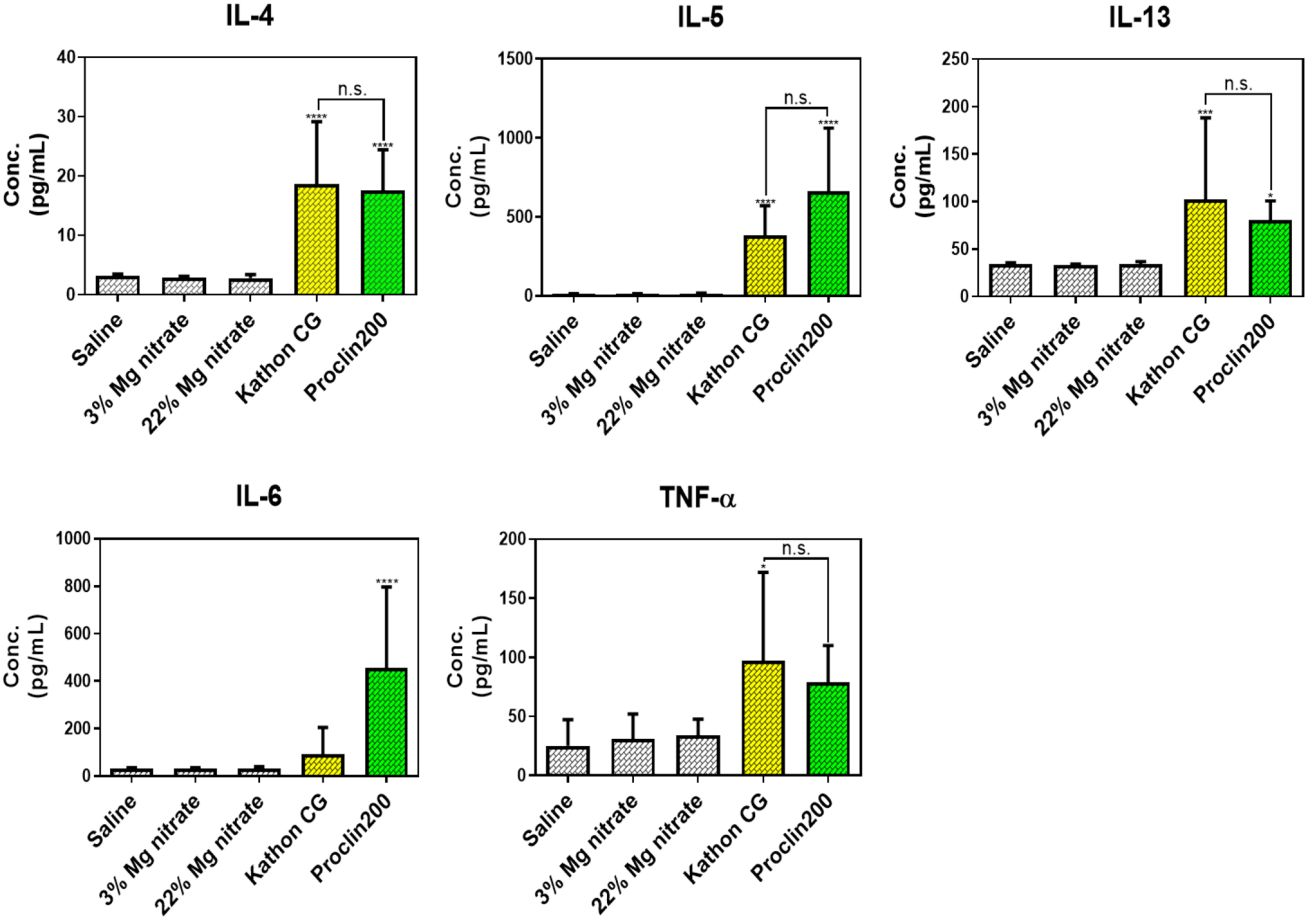
Measurement of the inflammatory cytokine levels in BALF by enzyme immunoassay of IL-4, IL-5, IL-13, IL-6, and TNF-α. The results were expressed as the mean ± SD for each group. *p < 0.05, ***p < 0.001 and ****p < 0.0001 vs. the saline control group. n.s., not statistically significant

Results showed that IL-4, IL-5, IL-13, and TNF-α levels were significantly increased in the BALF of mice exposed to both Kathon CG and Proclin 200 (Fig. 5). The level of IL-6 tended to increase in the Kathon CG-instilled mice, but there was no statistical significance.

Except for the level of IL-6, the expression changes of IL-4, IL-5, IL-13, and TNF-α were similar in the Kathon CG- and Proclin 200-instilled groups.

## Discussion

Humans are exposed to numerous household chemicals in the form of mixtures daily. However, most chemical toxicity assessments are designed to assess the health effects of exposure to individual chemicals. Although various chemicals such as stabilizers and additives exist as a single product in the form of mixtures, studies on combined toxicity are limited.

The mixture of CMIT/MIT is inherently unstable and commercial products require the addition of a stabilizer. The most widely used stabilizer for mixtures of CMIT/MIT is magnesium nitrate, which is highly hygroscopic and is used as a stabilizer and desiccant (ECHEMI, https://www.echemi.com/cms/719825.html). The highly hygroscopic property of magnesium nitrate changes the aerosol properties in the air when it is sprayed through a humidifier, and thus can affect toxicity. In this study, we focused on the effects of magnesium nitrate on CMIT/MIT-induced respiratory toxicity rather than the aerosol properties. This is the first animal study on respiratory toxicity due to the stabilizer magnesium nitrate and the effects of magnesium nitrate on the respiratory toxicity of CMIT/MIT under commercial product content conditions.

To determine the respiratory effect according to the magnesium nitrate content, the active ingredient, CMIT/MIT, was instilled at the same dose. The dose of the CMIT/MIT in Kathon CG and Proclin 200 was set to 1.14 mg a.i./kg, and the doses and dosing schedules were set the same as those causing lung damage due to Kathon CG reported previously [5].

Observation of general symptoms among the mice that received the Kathon CG and Proclin 200 showed wheezing or irregular respiration after ITI dosing, and we suggest that both CMIT/MIT-containing products have a direct effect on the respiration of the animals. Previous studies using impulse oscillometry (IOS) revealed that significant peripheral airway dysfunction was found in children with high levels of inhalation exposure to a mixture of CMIT/MIT [13]. Wheezing is the symptomatic manifestation of respiratory disease, including airway obstruction and airway dysfunction [14], and the published data suggested that wheezing was related to spirometry and IOS [15]. It can be suggested that the wheezing that appears after Kathon CG and Proclin 200 instillation is a symptom of airway dysfunction induced by CMIT/MIT. Meanwhile, there were no differences in the frequency and severity of respiration symptoms between the Kathon CG and Proclin 200 groups, which indicated that both Kathon CG and Proclin 200 induced similar respiratory dysfunction.

The mice that received Kathon CG and Proclin 200 showed significant body weight loss compared with those in the control group. The loss of body weight was continuously observed throughout the dosing period in both the Kathon CG and Proclin 200 exposed groups, and such results are caused by the induction of systemic or organ toxic effects following repeated exposure to CMIT/MIT. It was known that the body weight loss observed during experiments could be used as an early marker of organ toxicity [16].

Macroscopic findings showed red or pale discoloration of lungs in all the mice that received Kathon CG and Proclin 200, and there was no difference in the discoloration effect between those two groups. Pulmonary discoloration is a major hallmark of tissue injury, which is suggestive of pulmonary hemorrhage in lung injury models like acute respiratory distress syndrome [17]. The observed findings indicated both Kathon CG and Proclin 200 induced tissue injury in the lungs.

The mice that received Kathon CG and Proclin 200 showed a significant increase in relative lung weight. The increase in the weight of the lungs could be attributed to the infiltration of inflammatory cells into the lungs, facilitated by increased vascular permeability owing to lung injury [18]. A previous study determined that the change in lung weight, primarily the increase, was directly associated with the incidents of histopathological findings and with the maximal grades of all inflammatory lesions, the grades of alveolar macrophages, or the grades of perivascular inflammation [19]. Meanwhile, there was no difference in changes in the relative lung weight between the Kathon CG and Proclin 200 groups, which indicates that both Kathon CG and Proclin 200 induced similar lung injury due to CMIT/MIT.

Histopathological findings showed that eosinophils infiltration, granulomatous inflammation, inflammatory cell infiltration, mucous cell hyperplasia, and pulmonary fibrosis were commonly observed in the Kathon CG-exposed and Proclin 200-exposed mice, with similar frequency and severity in the two groups.

The BAL analysis results showed that the increased percentage of inflammatory cells, the increased cell count, the pattern of the eosinophils-dominated inflammatory response, and the increased Th2 cytokine level were similar between the Kathon CG and Proclin 200 groups.

Previous studies in our research group had identified eosinophil-mediated and Th2 cell-mediated fibrotic lung injuries caused by Kathon CG [5]. The present study confirmed that the cytological and histological patterns of lung injury, except for BW change, were similar between the Kathon CG and Proclin 200 groups. Although the change in BW was significantly greater in the Procline 200-instilled group than in the Kathon CG-instilled group, no significant differences in the number of inflammatory cells in the BAL and the pattern and severity of histopathological injuries between the two groups were found. The differences in BW changes between the two groups were not significantly related to respiratory toxicity. Further studies are needed to determine whether the differences in BW changes between the two groups are related to other forms of toxicity.

Accordingly, we suggest that magnesium nitrate concentration may not affect CMIT/MIT-induced lung injury. In addition, the present study showed that no respiratory effects were induced by intratracheal instillation of magnesium nitrate at the dose contained in the product. Although the results were negative, they gave us a better understanding of the toxicity of CMIT/MIT-containing mixture products and allowed us to focus more on the active substance in our humidifier disinfectant research.

This study has some limitations. Intratracheal instillation is not a satisfactory alternative to inhalation and may result in varied distribution of substances in the lungs. Previous studies have reported that inhalation of magnesium nitrate may cause respiratory irritation but no respiratory effects were observed with intratracheal instillation of magnesium nitrate [7]. The region of the respiratory tract affected by particle deposition largely depends on the particle properties, thereby affecting respiratory toxicity [20]. Magnesium nitrate-containing substances show different particle sizes and densities as they absorb water vapor from the humid environment of the human respiratory tract. Accordingly, by inhalation, the distribution and behavior of CMIT/MIT in the lungs may be affected depending on the magnesium nitrate content, in turn affecting toxicity. Therefore, additional inhalation studies are needed to confirm the respiratory toxicity of magnesium nitrate itself, and the distribution and toxicity differences for CMIT/MIT in the lungs according to the magnesium nitrate concentration. It is necessary to study not only the combined toxicity of active ingredients and additives but also the combined effect of mixtures of actual household chemicals.

In conclusion, a comparison of the respiratory effects caused by repeated ITI of two CMIT/MIT-containing products with different magnesium nitrate contents (Kathon CG containing 22% magnesium nitrate and Proclin 200 containing 3% magnesium nitrate) in the C57BL/6 mice suggested that the cytological and histological patterns of lung injury were similar between the two products, and accordingly, we suggest that magnesium nitrate concentration may not affect the CMIT/MIT-induced lung injury.

## Data Availability

The datasets generated during and/or analyzed during the current study are available from the corresponding author on reasonable request.

## Abbreviations

BAL: Bronchoalveolar lavage
BALF: Bronchoalveolar lavage fluid
BW: Body weight
CMIT: Chloromethylisothiazolinone
ELISA: Enzyme-linked immunosorbent assay
HD: Humidifier disinfectant
HDLI: Humidifier disinfectant-associated lung injury
H&E: Hematoxylin and eosin
IOS: Impulse oscillometry
ITI: Intratracheal instillation
MIT: Methylisothiazolinone
MT: Masson’s trichrome
PAS: Periodic acid-Schiff
PGH: Oligo(2-(2-ethoxy)ethoxyethyl guanidinium chloride
PHMG-P: Polyhexamethylene guanidine phosphate
Th2: Type 2T helper cells
